# Hepatitis B virus-infection related cryoglobulinemic vasculitis. Clinical manifestations and the effect of antiviral therapy: A review of the literature

**DOI:** 10.3389/fonc.2023.1095780

**Published:** 2023-02-07

**Authors:** Cesare Mazzaro, Riccardo Bomben, Marcella Visentini, Laura Gragnani, Luca Quartuccio, Francesco Saccardo, Marco Sebastiani, Davide Filippini, Gianfranco Lauletta, Giuseppe Monti, Valter Gattei

**Affiliations:** ^1^ Clinical and Experimental Onco-Haematology Unit, Centro di Riferimento Oncologico di Aviano (CRO) IRCCS, Aviano, Italy; ^2^ Department of Translational and Precision Medicine, Sapienza University of Rome, Rome, Italy; ^3^ Centro Manifestazioni Sistemiche da Virus Epatitici, University of Florence, Firenze, Italy; ^4^ Rheumatology Clinic, University of Udine, Udine, Italy; ^5^ Department of Internal Medicine, Saronno General Hospital, Saronno, Italy; ^6^ Rheumatology Unit, Department of Surgery, Medicine, Dentistry and Morphological Sciences with Transplant Surgery, Oncology and Regenerative Medicine Relevance, University of Modena and Reggio Emilia, Modena, Italy; ^7^ Department of Rheumatology, Niguarda Hospital, Milano, Italy; ^8^ Department of Biomedical Sciences and Human Oncology, Section of Internal Medicine and Clinical Oncology, Liver Unit, University of Bari Medical School, Bari, Italy

**Keywords:** hepatitis B virus, HBV extra-hepatic manifestations, HBV-related glomerulonephritis, HBV-related cryoglobulinemia, HBV-related vasculitis, entecavir, tenofovir

## Abstract

**Objective:**

Hepatitis B virus (HBV) infection causes chronic hepatitis, cirrhosis, and hepatocellular carcinoma. Furthermore, about 20% of the patients develop extrahepatic manifestations such as cryoglobulinemic vasculitis (CV), polyarteritis nodosa, non-rheumatoid arthritis, glomerulonephritis and non-Hodgkin lymphoma. This review analyzed literature data on clinical manifestations of HBV-related CV and the impact of antiviral therapy with analoques nucleotide.

**Methods:**

A PubMed search was performed to select eligible studies in the literature, up to July 2022.

**Results:**

Some studies have analyzed clinical manifestations in HBV-related CV and have investigated the role of antiviral therapy with nucleotides analogues (NAs). Clinical manifestations of CV vary from mild to moderate (purpura, asthenia and arthralgias) to severe (leg ulcers, peripheral neuropathy, glomerulonephritis, and non-Hodking lymphoma). NAs therapy leads to suppression of HBV-DNA; therefore, it is capable of producing clinical response in the majority of patients with mild to moderate symptoms.

**Conclusion:**

Antiviral therapy with NAs is the first choice for HBV suppression and control of mild to moderate disease. In severe vasculitis (glomerulonephritis, progressive peripheral neuropathy and leg ulcers), rituximab alone or with plasma-exchange is always indicated in combination with antiviral therapy.

## 1 Introduction

Hepatitis B virus (HBV) infection leads to about 250 million infected people worldwide. It is a hepatotropic virus, which in most cases evolves into cirrhosis and hepatocellular carcinoma causing an estimated 887,000 deaths each year (1). Extraepatic manifestations may occur in acute or chronic HBV infections, which in part have long been underestimated (2). HBV is also a lymphotropic virus; in fact, approximately 20% of patients may develop extrahepatic manifestations, such as cryoglobulinemic vasculitis (CV), polyarthritis nodosa, glomerulonephritis and non Hodking lymphoma (NHL) (3). Extrahepatic manifestatìons have a role in patients’ morbidity, quality of life, and mortality. Some studies have analized clinical manifestations in HBV-related CV (4–6). Pegylated interferon-alpha (PEG-IFN-alpha) or nucleotide analogues (NAs) are the current treatment; however, these often require treatment throughout the patient’s life because they do not eradicate the virus (7). Nonetheless, HBV-DNA suppression generally improves extraepatic manifestations. Some studies on the topic have shown a comparable NAs antiviral efficacy in HBV-related CV, in addition to a link between antiviral response and CV improvement (2, 4–6). This review focuses on clinical manifestations and on the role of NAs therapy in HBV-related CV.

## 2 HBV-related cryoglobulinemic vasculitis

Cryoglobulinemia has been defined as the presence in serum of immunoglobulins, which precipitate when the temperature drops below 37°C and redissolve when rewarmed (8, 9). Type I cryoglobulinemia includes a single monoclonal immunoglobulin IgM, IgG or IgA. It is associated with multiple myeloma, Waldenstrom’s disease, and NHL. In types II and III, referred to as mixed cryoglobulinemia (MC) (8), the cryoglobulins are immunocomplexes composed of polyclonal IgG with monoclonal or polyclonal IgM with rheumatoid factor activity. Until the 90’s, MC was associated with HCV infection in nearly 90% of cases (10, 11) while 10% of them were secondary either to lymphoproliferative disorders or rheumatologic diseases (12). The role of HBV as aetiologic agent of mixed cryoglobulinemia was firstly suggested by Levo et al. (13) more than 40 years ago. The authors conducted a pivotal study on 30 patients with essential mixed cryoglobulinemia suggesting that viral infection can be a main driver of the immunocomplex vasculitis. Monti et al. (14) retrospectively analyzed a cohort of 717 subjects with essential cryoglobulinemia followed by the Italian Group for the Study of Cryoglobulinemia (GISC) reporting a 5.5% prevalence of HBsAg positivity. Subsequently, Ferri et al. (15) evaluated 231 patients with MC, observing a 1.8% prevalence of HBsAg. All these data have been recently reviewed by Cacoub et al. (2). HBV-DNA and a high rate of HBsAg or anti HBc antibody are found in HBV-related CV (6). MC is a systemic vasculitis involving the small vessel caused by the precipitation of circulating cryoglobulin (16). MC symptoms often include purpura, asthenia, and arthralgia (Lo Spalluto Meltzer triade). Sensitive-motor peripheral neuropathy is a common manifestation, and glomerulonephritis can also be present (16). Membranoproliferative glomerulonephritis is the main renal manifestation of MC. In rare cases, it evolves into low-grade NHL. Less frequently, CV has a more severe, life-threatening presentation such as heart vasculitis, gastrointestinal vasculitis, or central nervous system involvement (9). The prevalence of chronic HBV infections in CV has now been documented in various case series over the past 20 years in Europe (manly Italy and France), with estimates ranging from 0.5% to 5.5% of cases (14, 15, 17, 18). HBV associated CV might be more common in Chinese patients (19). No studies so far have investigated CV incidence in HBV patients.

## 3 Main clinical manifestations of HBV-related CV

**Table 1** summarizes the main studies on HBV-related CV. Some clinical and epidemiological studies found in literature suggest a casual relationship of HBV with MC (**Table 1**) (4, 6, 20). Our analysis of the studies found in literature, on HBV-related CV, revealed that about 80% HBV-MC patients had chronic hepatitis while cirrhosis was present in 20% of them. The reported cases had mild–moderate clinical symptoms (palpable leg purpura, asthenia and arthralgias were present from 40% to 90% of cases (4, 6, 20). Purpura on the leg was palpable, orthostatic, and bilateral with recurrence during the winter season. Bilateral and symmetric joint pain, non-deforming and mainly involving knees and hands, usually characterize articular involvement. Skin ulcers may develop in about 20% of cases. Sicca syndrome and Raynaud’s phenomenon have been reported in about 20% of patients. Neurologic manifestations ranging from distal sensory to sensory-motor polyneuropathy -this latter involving about 30% of patients- may increase to as high as 70% of cases when electromyography is employed. Pain and asymmetric, subsequently symmetric, parsthesia is reported by patients. The motor neuropathy is sporadic, mainly affecting the legs, and usually occurring a few years after the sensory neuropathy. As in HCV-related CV, type I membrane-proliferative glomerulonephritis (MPGN) is the most frequent kidney manifestation. Nephrotic range of proteinuria and microscopic hematuria, often with evidence of renal insufficiency are common characteristics of HBV-MPGN. Kidney involvement emerges as an unfavorable prognostic factor (20, 21). Both aggressive and indolent B-cell NHL are observed in about 10% of the patients (6, 21).

**Table 1:** Mains studies on HBV-related cryoglobulinemic vasculitis.

	Boglione et al. (4)	Terrier et al. (18)	Li et al (19)	Mazzaro et al. (6)
Patients (N.)	(7)	(3)	(12)	(18)
Females/Males	3/4	2/1	4/8	10/8
Age, years, median (range)	60 (49-65)	49 (41-65)	47 (28-68)	59 (33-81)
Virological and biochemical features				
HBs Ag positive, n. (%)	7 (100)	3 (100)	10 (83)	18 (100)
HBV-DNA positive n. (%)	7 (100)	3 (100)	12 (100)	18 (100)
Type II/Type III	n.d.	3/0	3/9	17/1
Cryocrit, % median (range)	3.4 (2.5-6)	3 positive	n.d.	5 (1-70)
RF IU/mL, median (range)	n.d.	n.d.	694 (67-2,730)	350 (15-5,850)
C4 mg/dL, median (range)	n.d.	10 (1-25)	6.0	8 (2-28)
ALT U/L, median (range)	79 (12-638)	n.d.	44/10-102)	82 (12-638)
Creatinine mg/dL, median (range)	n.d.	n.d.	2.8 (0.0-9.8)	1.0 (0.6-1.3)
Clinical manifestations				
Purpura,n (%)	3 (43)	2 (67)	7 (58)	17 (94)
Asthenia, n (%)	0	0	3/35%)	16 (89)
Arthralgias	0	2 (67)	3(35)	14 (78)
Ulcer of the leg	2 (20)	0	0	5 (28)
Sicca syndrome	0	0	0	5 (28)
Raynaud’s phenomenon	0	0	0	3 (17)
Peripheral neuropathy	4 (57)	0	2 (17)	13 (72)
Chronic hepatitis	n.d.	3 (100)	n.d.	16 (89)
Liver cirrhosis	n.d.	0	n.d.	1 (6)
Glomerulonephritis	0	3 (100)	12(100)	2 (11)
Non-Hodgkin lymphoma	0	0	0	1 (6)

AST, aspartate aminotransferase; ALT, alanine aminotransferase;

GGT, gamma-glutamyl transferase; RF, rheumatoid factor.

n.d., not done.

## 4 Management of HBV-related CV

Although HBV-related CV guidelines for specific treatment are not available (22), it is generally accepted that the goal to obtain remission of extraepatic manifestations to prevent complication is reducing HBV replication to undetectable HBV-DNA (2). Vaccine compaigns and the highly efffective NAs therapy have led in the past 10-15 years to a decreasing prevalence of CV (2). Patients with chronic HBV-related CV are treated with antivirals regardless of severity of liver disease. Because of its infrequent occurrence, HBV-related CV is considered a rare disease; therefore, data regarding its management is limited. Treatment should be tailored according to CV severity. Treatment management of HBV-CV can follow the same approaches used for HCV-related CV. Current treatment include PEG-IFN-alpha or NAs. PEG-IFN alpha has the advantage of a limited therapy time (12 months) and slightly higher rates of HBsAg and HBeAg seroconversion, but it carries several contraindications and severe side effects, which discourage its use in HBV-related CV. PEG-IFN alpha has an ancillary role in HBV-related CV (23).

The antiviral therapy with NAs is the first choise for HBV suppression and control of a mild–to-moderate disease. Antiviral therapy could be administered in combination with glucocorticoids to control vasculitis recurrence, with plasma exchange to remove cryoglobulins in the serum, and with rituximab (anti CD-20) to eliminate B lymphocytes that produce cryoglobulins (6).

## 5 Antiviral treatment of HBV-related CV

**Table 2** describes the main studies on the use of NAs in the management of HBV-related CV (4–6, 18, 20). These studies have shown an association of viral suppression by NAs therapy with reduction of cryoglobulins, normalization of rheumatoid factor, and disappearance of purpura on the leg, asthenia, and arthralgia. These improvements persisted throughout antiviral therapy. In all patients, HBsAg remained positive. Unlike the virologic response, the clinical and immunological outcomes of antiviral therapy were less effective. In fact, during treatment, about 75% of cases showed an improvement or disappearance of signs and symptoms associated with CV. Manifestations such as purpura, asthenia, and arthralgia had clinical improvements in 50-90% of cases within 6-9 months since starting NAs therapy. On the other hand, an improvement of ulcers and peripheral sensory prevalent neuropathy during antiviral therapy was achieved on average by 50% of cases. In patients with peripheral severe sensory-motor neuropathy, no clinical improvement was generally observed after the suppression of HBV by NAs. These patients showed clinical and immunologic improvements with rituximab. The analyzed studies suggested that rituximab in combination with antiviral therapy should be considered in patients with severe peripheral neuropathy. In few patients with HBV-related glomerulonephritis, treatment with NAs improved creatinine levels and mildly proteinuria. (**Table 2**). In patients with glomerulonephritis with no clinical response to antiviral therapy, treatment with rituximab alone or associated with plasma-exchange has been shown to be effective in improving both immune and renal functions (**Table 2**) (6, 18, 20). In a recent report, one patient who received entecavir, low-dose rituximab, and plasma-exchange experienced renal function improvement and HBV-DNA suppression (6). A second patient affected by low-grade NHL who received antiviral therapy with tenofovir required subsequent chemotherapy and experienced partial hematological response (6). The third CV patient, after 60 months of therapy with entecavir, developed a cerebral diffuse large B-cell lymphoma. This patient died due to lymphoma progression despite chemotherapy (6). After suppression of HBV by antiviral therapy, disappearance of cryoglobulinemia was observed in about 60% of cases, normalization or decrease of rheumatoid factor in 50%, and normalization of serum C4 level in about 30%, and their persistence predisposes to probable relapses. Patients with HBV-related CV with purpura and nephropathy who were treated with rituximab in combination with NAs experienced a clinical remission (18, 20).

**Table 2:** Nucleotide analogues (NAs) therapy in patients with HBV-related cryoglobulinemic vasculitis, according to studies.

	Boglione et al. (4)	Terrier et a (17)	Mazzaro et al. (5)	Li et al. (19)	Mazzaro et al. (6)
***Patients N.***	***(7)***	***(3)***	***(7)***	***(9)***	***(18)***
**Laboratory** **Features, N.** **(%)**	Cryocrit median: 4% (2.5- 6.0%)	Cryocrit pos.: 3 (100) C4 median: 10 (1-25)	Type II: 7 (100) Cryocrit median: 3% RF median: 200 C4 median: 8	Type I: 3 (33); Type III: 6 (67), Cryocrit: 1900 mg/L; RF median: 82; C4 median: 5; Creatinine median: 2.2g/dl; Proteinuria median: 5.0 g/day	Type II: 17 (94); Type III: 1 (6); Cryocrit median: 4% (1-70); RF median: 181 (10-5850); C4 median: 9 (2-31); ALT median: 51 (21-638)
**Liver status, N. (%)**	Chronic hepatitis: 7 (100)	Chronic hepatitis: 3 (100)	Chronic hepatitis: 6 (86) Cirrhosis: 1 (14)	NA	Chronic hepatitis: 4 (22) Cirrhosis: 1 (6)
**Clinical Manifestation, N. (%)**	Purpura: 3 (43) Per. neuropathy: 4 (54) Skin ulcer: 2 (29)	Purpura: 2 (67) Arthralgias: 2(67) Glomerulonephritis: 3 (100)	Purpura: 7 (100) Arthralgias: 7(100) Ulcer on the leg: 1 (14)	Purpura: 4 (44) Arthralgias: 2 (22) Per. Neuropathy: 2 (22) Gastrointestinal vasculitis: 2 (22) Glomerulonephritis: 9 (100)	Purpura: 18 (100) Arthralgias: 11 (61) Ulcer on the leg: 3 (17) Sjogren S.: 5 (28) Per. neuropathy: 11 (61) Chronic hepatitis: 4 (22) Cirrhosis: 1 (6) ALT median:72 Glomerulonephritis: 1 (6) NHL: 2 (11)
**Antiviral agent** **Dose =** **Duration, Weeks (w), Month (m)**	Telbivudine 600 mg/day: 7 (100%) = 48 w	Lamivudine 100 mg/day: (1) Entecavir 0.5 mg/day: (2)	Entecavir: 5 = 48 m Adefovir:1 = 48 m Lamivudine: 1 = 48 m	Entecavir 7 = 16 m Lamivudine 2 = 12 m	Entecavir: 11 = 66 m Tenofovir: 6 = 67 m Lamivudine: 1 = 59 m
**SVR after NAs, N. (%)**	7 (100)	3 (100)	7 (100)	9 (100)	18 (100)
**Concomitant immune suppression, N. (%)**	—	PE + CS + RTX: 1 (33) PE + CYC+ CS + RTX: 1 (33)	CS alone:1(14)	CS alone: 3 (33) CS + CYC:1 (11) CS + PE + RTX: 1 (11) CS + PS + MMF: 1 (11)	Peg-IFN alone: 3 (38) CS associated Nas: 4 (22) PE associated NAs: 4 (22) RTX associated NAs:2 (11)
**Immune response, N. (%)**	Cryocrit median: 1% (0-2)	Cryocrit negative: 1 (33) Cryocrit positive: 2 (67)	Cryocrit median: 1% RFmedian:86 C4 median: 10 ALT median:20	Creatinina median: 1 mg/dl Proteinuria median:1.6 g/day	Cryocrit median:1% (0-14) RF median: 181 (10-5850) C4 median:7 (1-24) ALT median: 16 (12-34)
**Complete clinical response, N.**	Purpura: 3(43) Per. neuropathy: 2 (29)	Purpura: 2 (67) Athralgias: 2 (67) Glomerulonephritis: 1 (33)	Purpura: 7 (100) Arthralgias: 5 (71) Ulcer on the leg: 1 (14)	Purpura: 2 (22), Arthralgias: 2 (22), Per. neuropathy: 2 (22) Glomerulonephritis: 2(22)	Purpura: 14 (78) Arthralgias: 8 (73) Ulcex: 2 (67) Sjogren S.: 2 (40) Per. neuropathy: 6 (55)
**Partial clinical response, N. (%)**	——	–	–	Glomerulonephrirtis: 3 (33)	Glomerulonephritis: 1 (100)) NHL: 1 (50)
**No clinical response, N. (%)**	Per. neuropathy: 2 (29) Skin ulcer: 2 (29)	Glomerulonephritis: 2 (67)	Arthralgias: 2 (28)	Glomerulonephritis: 4 (44), dialysis in 4 pts, 2 (22) died	Purpura: 2 (11) Arthralgias: 3 (27) Neuropathy: 5 (45) NHL:1 (50)

GN, glomerulonephritis; CS, Corticosteroid; CYC, cyclophosphamide; RTX, Rituximab; MMF, mycophenolate; PE, plasma exchange; Per. Neuropathy, Peripheral Neuropathy.

NA, Not Available.

## 6 Discussion

In the context of patients with HBV infection, CV is a rare event, since it occurs in only 0.5%-5.5% of the patients (14, 15, 17, 18). Conversely, according to Lunel et al. (24), the prevalence of MC in patients with chronic HCV infection was 54%, and according to Adinolfi et al. (25) it was 47%, whereas only 27% was estimated to develop clinical signs of vasculitis (24). HBV is a lymphotropic virus and can induce cryoglobulinemia and B-NHL. Several epidemiological studies have shown a significant association between HBV, cryoglobulinemia and B-NHL. The pathogenic role that leads HBV chronic stimulation through many mechanisms to lymphoma has not been established yet. As antiviral treatments have been shown by some studies to be effective in a significant proportion of patients, they are usually introduced as the first-line treatment. Antiviral therapy is mostly based on mono-therapy with NAs, such as lamivudine, adefovir, entecavir, tenofovir, or telbivudine, which have given excellent results in viral suppression and concurrent good clinical response of vasculitis in HBV-related CV (4, 5, 20, 26, 27). Some studies have shown a correlation between HBV-DNA suppression with NAs therapy and reduction of cryoglobulins and improvement or disappearance of CV symptoms for the most part of patients (4–6, 20, 26).

A few studies reported some benefit of antiviral treatment with telbivudine and entecavir in patients affected by skin ulcers in HBV-associated CV (4–6). In these studies, improvement of peripheral neuropathy was obtained in few cases with tenofovir or entecavir alone. The other patients with debilitating peripheral neuropathy were treated with NAs in combination with plasmaferesis and subsequently rituximab to obtain disappearance of symptoms (6, 20). Some studies (6, 18) have demonstrated in very few cases the efficacy and safety of the anti-CD20 monoclonal antibody (rituximab) associated with NAs in HBV-associated cryoglobulinemic glomerulonephritis. These data appears to be relevant since renal involvement in CV is associated with a severe prognosis (6, 12, 18, 20, 22). A few cases with low-grade NHL who received antiviral therapy with NAs have required subsequent immunotherapy or chemotherapy as they did not experience hematological response; nonetheless, antiviral therapy should be recommended to patients with HBV-related CV complicated by low-grade NHL to eliminate chronic viral stimulation. Association of NAs with chemotherapy is recommended in patients who relapse or do not respond to antiviral therapy. Chemotherapy associated with NAs therapy is recommended in HBV-associated high grade NHL (22). The reactivation of the hepatitis B virus following chemotherapy or immunotherapy (rituximab or steroid) is a significant issue in patients with CV and non-Hodgkin’s lymphoma (NHL). Increased risks of liver failure and death in extremis cases due to an HBV reactivation have been documented in a number of previous research studies (28, 29). The use of prophylactic administration of lamivudine or entecavir to prevent HBV reactivation, before initiation of immunosoppression is essential (28, 29). Antiviral therapy with NAs should be maintained for an indefinite period (5), and only after persistent HBsAg/AntiHBsAb seroconversion and undetectable HBV-DNA it can be discontinued (7).

## 7 Conclusions

**Figure 1:**
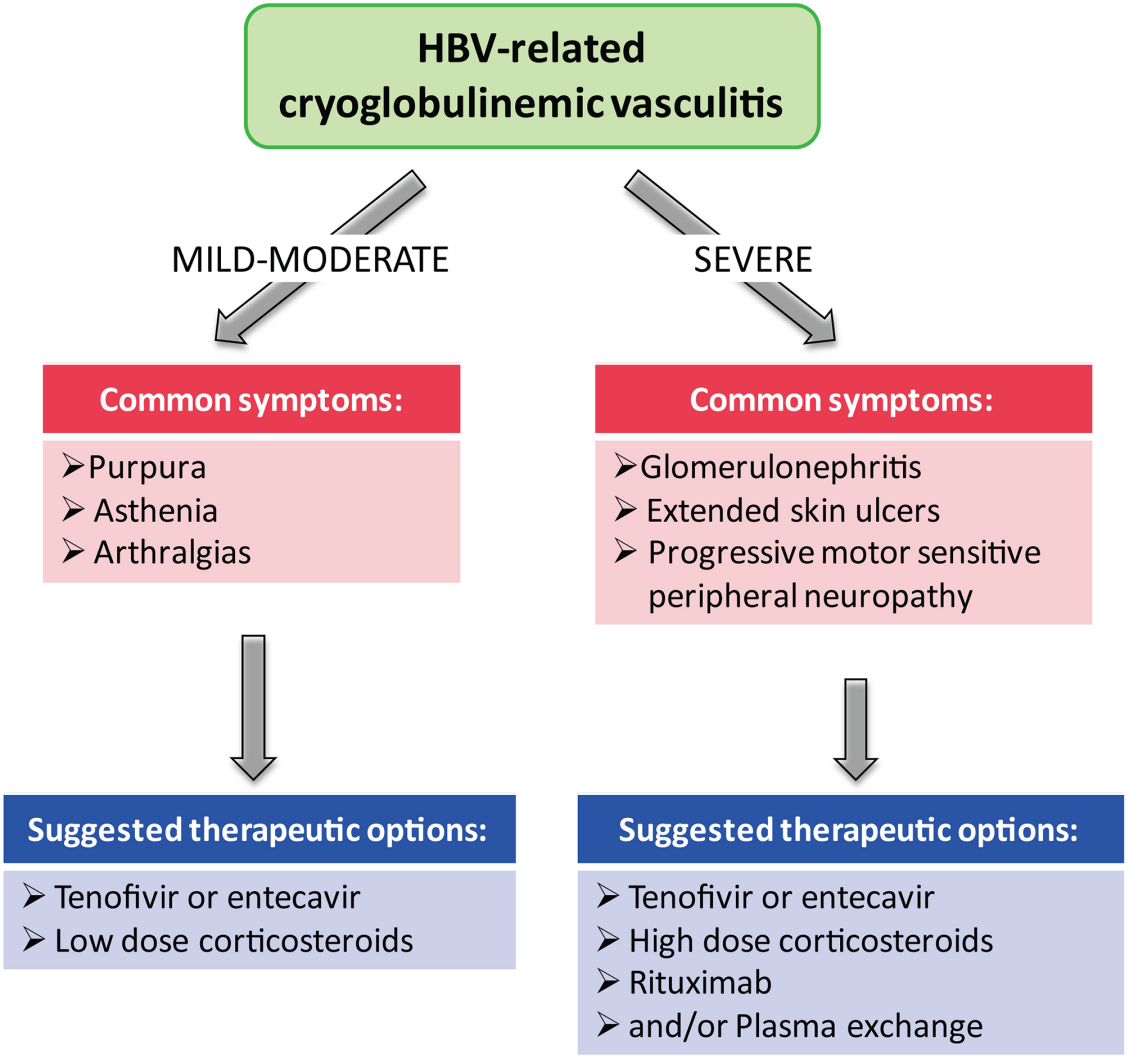
Therapeutic strategies in HBV-related cryoglobulinemic vasculitis.

Presently, the best therapy, in HBV-related CV, is generally based on monotherapy with NAs, which can induce, in most patients with mild-to moderate manifestations (purpura, asthenia, and arthralgias), a complete viral suppression of HBV-DNA and give good clinical response (22) (**Figure 1**). In case of non responders or relapses and in severe CV (skin ulcers, debilitating peripheral neuropathy, and nephropathy), a second-line treatment with rituximab alone or associated with plasma exchange may be added to antiviral therapy (**Figure 1**). Another important goal of antiviral therapy is the early suppression of HBV-DNA to avoid organ complications and appearance of lymphoproliferative disorders (22).

## Author contributions

Conceptualization and methodology: CM and RB; software: CM and RB; validation: CM, RB, and LQ; investigation: CM, MV, LG, LQ, FS, MS, DF and GL; data curation: CM; writing the original draft: CM, RB, LQ, MV, GM, LG, and VG. All authors contributed to the article and approved the submitted version.
